# Synthesis of Poly(glycerol butenedioate)—PGB—Unsaturated Polyester toward Biomedical Applications

**DOI:** 10.1021/acsomega.2c01934

**Published:** 2022-07-15

**Authors:** Michał Wrzecionek, Krzysztof Kolankowski, Agnieszka Gadomska-Gajadhur

**Affiliations:** Faculty of Chemistry, Warsaw University of Technology, 3 Noakowskiego Street, Warsaw 00-664, Poland

## Abstract

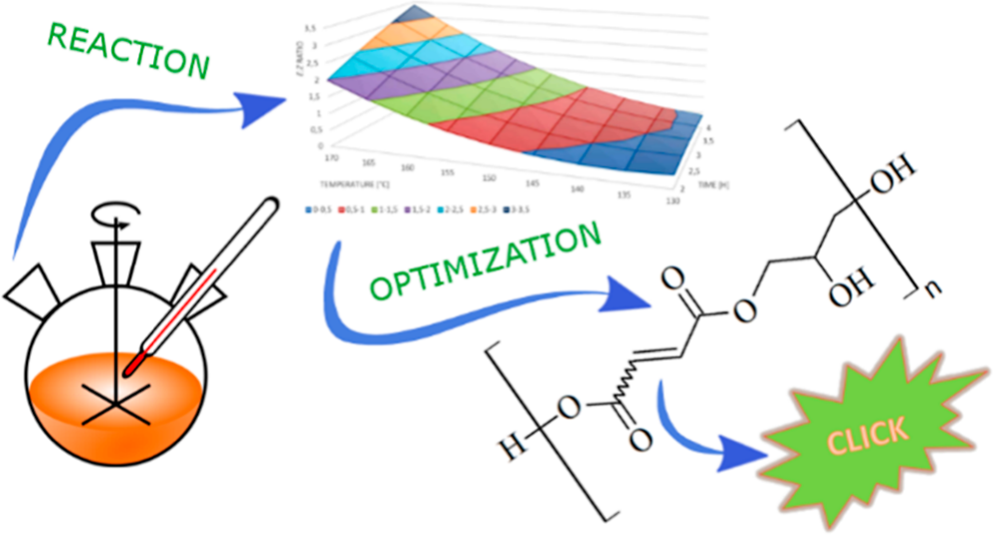

A new polyester poly(glycerol butenedioate) (PGB) was obtained in the bulk polycondensation of glycerin and maleic anhydride. Glycerol polyesters are new biomaterials commonly used in tissue engineering. PGB, containing the α,β-unsaturated moiety, could be very interesting due to potential modifications such as additions or oxidation. Such modifications are not possible on the heretofore known glycerol polyesters due to their structure without α,β-unsaturated moieties. In this work, the developed process was optimized by applying the design of experiments. The optimization criterium was the minimization of the E/Z isomer ratio. Applying the two-stage process, the *E*/*Z* isomer ratio was reduced from 5.5 to 0.5 compared to the one-stage process. The degree of branching was also reduced from 17 to 9%, as well as the degree of esterification from 0.89 to 0.72. The obtained structure can be used in modifying or cross-linking via Michael additions.

## Introduction

Nowadays, biomedical engineering is developing very rapidly. Scientific studies concerning scaffolds,^1−8^ drug delivery systems (DDSs),^9−16^ new medical devices,^17^ and implants^4,18^ are becoming more and more popular. Most often, these products are made of metallic, ceramic, or polymeric materials.^19^

Among the many polymers used for manufacturing biodegradable implants, scaffolds, and biomedical devices, polyesters are used most commonly. The most popular are polylactide,^11,17,20−23^ polyglycolide,^24−26^ and poly(ε-caprolactone).^5,27−30^ Polyesters of glycerin also exhibit high potential. This triol is a compound present in many living creatures and is commonly used in the pharmaceutical and cosmetic industry because of its safety for human health.^31,32^ The most important polyester of glycerin is poly(glycerol sebacate), which was mainly designed for manufacturing scaffolds and DDSs.^10,21,33,34^

The interest of polyesters of glycerin is connected to its low price and good availability. During the period 1999–2009, its production grown over 10 times and its price decreased ca. 5 times.^35,36^ Accordingly, scientists have developed many new technologies for glycerin usage in obtaining small molecular compounds, such as epichlorohydrin, methanol, or propylene glycol.^37^ The other example of glycerin application is polymer science.^31,38^ Until now, besides poly(glycerol sebacate), there are reports about other polyesters of glycerin in the literature, that is, poly(glycerol succinate),^31,33,39−43,71^ poly(glycerol adipate),^33,43−48^ poly(glycerol azealate),^39,42,49^ poly(glycerol iminodiacetate),^39^ poly(glycerol glutarate),^33,42^ poly(glycerol dodecanediate),^33^ poly(glycerol phthalate),^50−52^ poly(glycerol isophthalate),^33^ poly(glycerol terephthalate),^33^ poly(glycerol 2,5-furandicarboxylate),^53^ and poly(glycerol *cis*-octadec-9-ene-1,18-diate).^54^

The functionality of glycerin equals *f* = 3, so polyesters derived from it may have a different topology. Dendrimers are the most regular. They exhibit low viscosity, low polydispersity, and a high number of terminal groups^55,56^ and may be used as DDSs.^40^ Unfortunately, their synthesis has many steps during which the potentially dangerous compounds need to be used.^40,56^ Hyperbranched polyesters exhibit less regular topology. Their degree of branching is in the range of 40–60%.^57^ They are similar to dendrimers, and their synthesis is less complicated. Additionally, there is no need to use dangerous compounds such as benzaldehyde, which block the terminal groups in glycerin molecules during the synthesis of dendrimers.^39,41,42,44,46^ Polymers exhibiting low degree of branching (DB < 5%) are recognized as linear. The boro-organic catalysts^33^ or enzymes (i.e., *Candida Antarctica* lipase B)^43,48,54^ must be used for their synthesis. However, they are unsuccessful in obtaining linear polyesters of short dicarboxylic acids (i.e., succinic or glutaric).

The properties of polyesters can be chemically modified due to the presence of unreacted hydroxyl or carboxyl groups. It is very important for obtaining materials for special uses such as DDSs or for regeneration of a specific tissue. In the case of unsaturated polyesters, the presence of double bonds in macromolecular chains can also be utilized for cross-linking or modifying, that is, by radical reactions.^58^ Modification through unsaturated bond reaction is often easier and more precise to do. Also, it keeps the side and end functional groups intact. The commonly used monomers for obtaining unsaturated polyesters are isomers of butenedioic acid, which are fumaric acid and maleic acid, or their derivatives.

Maleic anhydride is one of the most often used because of its high reactivity, and moreover, it converts into *E*-isomer very easily after the ring opening. This phenomenon is used to synthesize poly(1,2-propanediol fumarate),^59,60^ which is applied in tissue engineering due to its biocompatibility, biodegradability, and physicochemical properties similar to bone tissue.^61−63^ Using proper catalysts and lower temperature can limit the isomerization of double bonds and allow to obtain a statistic copolymer—poly(1,2-propanediol maleate-*co*-fumarate). This material is also used in biomedical engineering.^61,64^ In the literature, there is only a little information about the synthesis of the copolymer, poly(glycerol succinate-*co*-maleate), but without its characterization.^65−67^

**Scheme 1:**
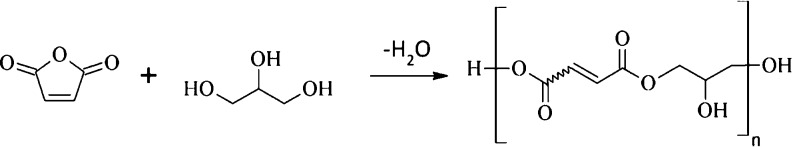
Polycondensation of Maleic Anhydride and Glycerine to PGB

We decided to obtain a new polyester, poly(glycerol butenedioate) (PGB), using maleic anhydride and glycerine as monomers in bulk polycondensation (Scheme 1). Moreover, we wanted to design the synthesis in order to obtain the best structure for further double-bond reactions. For this reason, we decided to limit isomerization and branching. This is to ensure better reactivity and steric availability of the α,β-unsaturated moiety.

## Materials and Methods

### Materials

Commercially available reagents (glycerin 99% and maleic anhydride 98%, Sigma-Aldrich) were used without further purification.

### Reactor Equipment

In this study, the Mettler Toledo MultiMax parallel reactor system was used. A Hastelloy 50 mL reactor equipped with a mechanical stirrer, a Pt100 temperature system, and a reflux condenser were used. For reducing the pressure, a vacuum ejector was used. A desirable pressure was set and controlled with the use of a Buchi B-720 Vacuum Controller.

### One-Step Synthesis Procedure

Maleic anhydride and anhydrous glycerin were mixed at a molar ratio of 0.8, heated to 150 °C, and stirred at 200 rpm for 3 h. Then, the temperature was changed to 130 °C. After another 15 min, the pressure in the reactor was reduced (500 mbar) in order to collect the water. The process was continued for 2 h. The product was analyzed without further purification.

### Two-Step Synthesis Procedure

Maleic anhydride and anhydrous glycerol were mixed at a molar ratio of 1:2.5, heated to 150 °C, and stirred at 200 rpm for 3 h. Then, according to the Box–Behnken plan (Table 4), an appropriate amount of maleic anhydride was added to the mixture, and the temperature was changed or maintained for 15 min. After another 15 min, the pressure in the reactor was reduced (500 mbar) to collect the water which was produced. The process was continued for several hours according to the Box–Behnken plan (Table 4). The products were analyzed without further purification.

### Spectral Analyses

IR spectra were obtained using a BRUKER ALPHA II Platinum ATR spectrometer (averaged 32 scans in the range 400–4000 cm ^–1^). NMR spectra were obtained using an Agilent spectrometer (400 MHz). Approximately, 150–200 mg of PGB was dissolved in deuterated dimethyl sulfoxide (DMSO) (1 mL, 99.8%, Deutero) for 24 h, and then the solution was transferred to an NMR tube. The degree of branching was calculated based on methine proton signals in accordance with the Frey method.^68^

### Titration Analyses

#### Acid Number (*AN*)

The sample (0.2–0.3 g) was weighed, and then 25 mL of MeOH and three to four drops of thymol blue were added. The sample was titrated with a 0.1 M aqueous solution of NaOH until the color changes from yellow to blue. Simultaneously, the blank test was conducted. The analysis was carried out in three replications. The standard error did not exceed 5%. The acid value (*AV*) was calculated according to the following formula
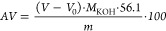
where *AV*—acid value (mg/g sample), *V*_0_—volume of 0.1 M KOH used to titrate the blank test, *V*—volume of 0.1 M KOH used to titrate the sample, *M*_KOH_—KOH titer used for titration (0.1 M), and *m*—mass of the sample.

#### Ester Number (*EN*)

The sample (0.2–0.3 g) was weighed. Methanol (15 mL) and 20 mL of 0.1 M of aqueous solution of KOH were added. It was heated in boiling water under a reflux condenser for 1 h. Upon cooling the solution, the excess of added KOH 0.1 M was titrated with hydrochloric acid in the presence of phenolphthalein. Simultaneously, the blank test was conducted. The analysis was carried out in three replications. The standard error did not exceed 5%. The ester value (*EV*) is calculated according to the following formula
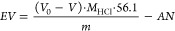
where *EV*—ester number (mg/g sample), *AN*—acid number, *V*_0_—volume 0.1 M HCl used to titrate the blank test, *V*—volume of 0.1 M HCl used to titrate the sample, *M*_HCl_—HCl titer used for titration (0.1 M), and *m*—mass of the sample.

#### Degree of Esterification

The degree of esterification (*DE*) was calculated on the basis of the following formula
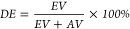
where *DE*—degree of esterification, *AV*—acid value, and *EV*—ester value.

All used titration methods are in accordance with our previous works.^21,69,71^

### Calculations

All calculations were performed with the use of MS Excel (2205 version) and Solver addon. All statistical data is presented in the Supporting Information (significance of coefficients of each equation, included coefficient estimate, standard error, the critical value for the *t*-Student test, the value of the *t*-Student test for each coefficient, and significance assessment). The DoE method was used to create the experimental matrix, while the RSM method was used to calculate the model.

## Results

### Preliminary Studies

Variants with and without collecting water, as well as one-step and two-step reactions, the total mass of anhydride divided and added in the first and second step of the reaction, were tested. A detailed description of the preliminary reactions is provided in the Supporting Information. Consequently, we decided to carry out the two-step reaction without water collection in the beginning and later to reduce the pressure to remove water from the reactor. Also, such a process allows limiting the isomerization of double bonds by reducing the residence time of maleic anhydride in the reactor. The anhydride-to-glycerin molar ratio was 2:5 in the first step. A high molar excess of glycerin was meant to guarantee a fast reaction of anhydride with glycerin, leading to short oligoesters with a low degree of branching, terminated mostly with glycerin molecules. To determine the moment of adding the next portion of anhydride, the acid value was controlled during the first step. Halved reduction of acid number was observed after 18 min, the reaction speed reduced drastically after 25 min and after 2 h remained almost constant heretofore. We decided to start the second step when the acid number became 50 [mgKOH/g], which happened after 3 h. In the beginning, the acid number was 339 [mgKOH/g]. The properties of the first-step reaction product are presented in Table 1.

**Table 1:** Properties of the First-Step Product

*E/Z*	DE	DB
0.71	0.89	9%

**Figure 1:**
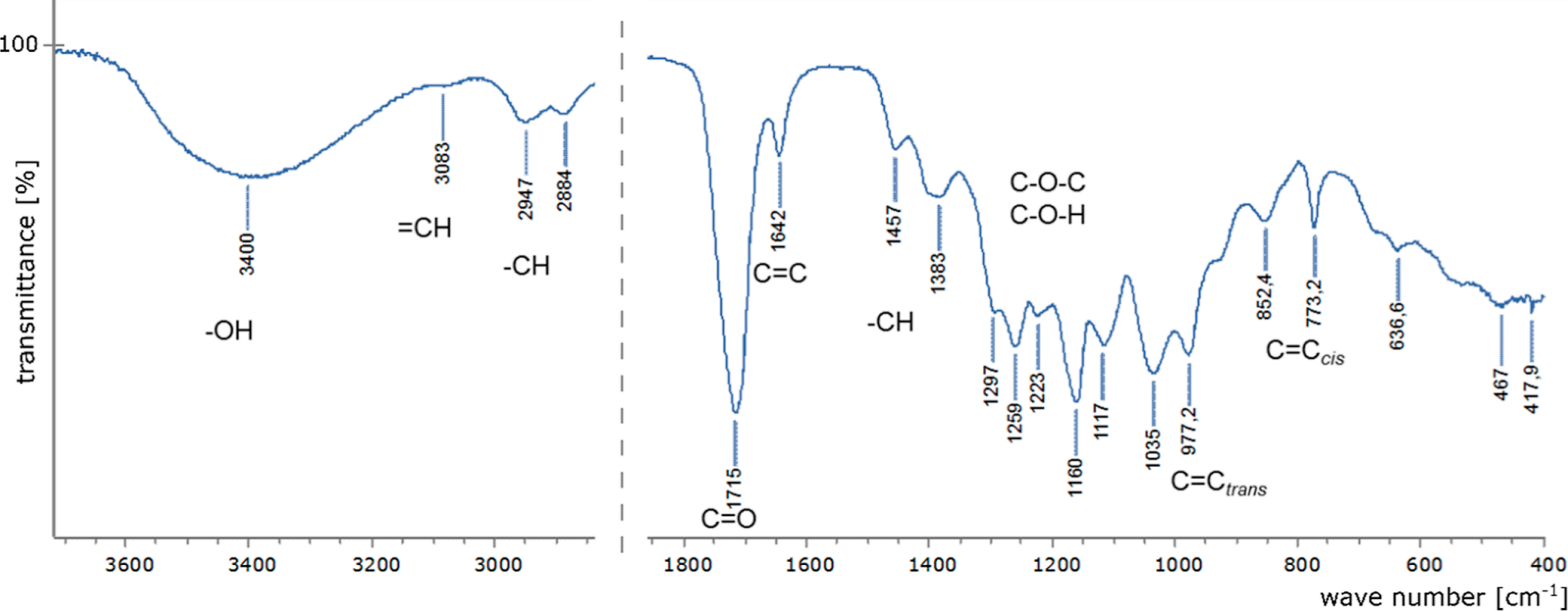
FTIR spectra of PGB.

After the first step, the anhydride was added to obtain a 1:1 ratio of the anhydride to glycerin. The synthesis was continued under reduced pressure (ca. 200 mbar) for 3 h, and the product was obtained in the form of a yellow sticky resin. The polyester structure was confirmed by FTIR spectroscopy (Figure 1), and the following bands were detected: a broad band of stretching vibrations in O–H bonds present in glycerol and carboxylic acids (ν = 3200–3600 cm^–1^), two bands of antisymmetric and symmetric stretching vibrations in C_sp3_-H (ν_as_ = 2950 cm^–1^ and ν_s_ = 2850 cm^–1^), an intense, sharp band of stretching vibrations in C=O bonds conjugated with C=C bonds present in unsaturated esters (*ν* = 1715 cm^–1^), a weak, sharp band of stretching vibrations in C=C bonds (*ν* = 1640 cm^–1^), a weak, sharp band of bending vibrations in C_sp3_-H bonds (*ν* = 1450 cm^–1^), a few intense bands of stretching vibrations in C–O–C and C–O–H bonds characteristic for α,β-unsaturated esters and alcohols (*ν* = 1300–1000 cm^–1^), a weak band of stretching vibrations in C–O–C bonds characteristic for ethers (*ν* = 1112 cm^–1^), and bands of bending vibrations in C_sp2-H_ bonds characteristic for, respectively, *E* and *Z* alkenes (ν_E_ = 973 cm^–1^ and ν_Z_ = 777 cm^–1^).

NMR spectroscopy was the main method for structure determination. With the use of one- and two-dimensional NMR experiments, the signal characteristics of unsaturated bond protons and glycerin methine protons were identified. The chemical shifts of each of them are presented in Table 2. The spectra are shown in the Supporting Information.

**Table 2:** Chemical Shifts in ppm of Characteristic Protons in PGB Structure (1H NMR, DMSO-*d*_6_)

unsaturated bond protons	acid	monoester	polyester
isomer *Z*	6.25	6.30–6.47	6.47–6.57
isomer *E*	6.63	6.65–6.67	6.80–6.88
methine protons	triglyceride	1,2-digliceride	1,3-diglyceride
	5.25–5.55	4.86–5.20	3.84–4.22
	1-monogliceride	2-monogliceride	
	3.58–3.72	4.73–4.86	

### Optimization

The second step of the reaction was optimized by using the Box–Behnken plan. This design was chosen because it allows the analysis of three variables on three levels and the modeling of the relationship by using a second-order polynomial, taking into account interactions. Moreover, in this case, only 15 experiments have to perform. The criterium of the optimization is to minimalize *E/Z* with *DB* less than 15%. Three variables were chosen: total anhydride/glycerin ratio, second-step reaction temperature, and second-step reaction time. The range of each tested variable is shown in Table 3. As output variables, the *E*/*Z* isomer ratio, the *DE*, and the degree of branching were measured.

**Table 3:** Area of the Experiment and the Limit Values

		the area of the experiment			
variable	BLV	(−1)	(0)	(+1)	ULV
*x*_1_—maleic anhydride/glycerin ratio	0.4	0.8	1.0	1.2	1.6
*x*_2_—the temperature [°C]	53	130	150	170	180
*x*_3_—the reaction time [h]	0.1	2	3	4	24

BLV—bottom limit value and ULV—upper limit value

Due to the use Box–Behnken plan, the three-factorial three-level plan, only 15 experiments have to be performed for mathematical modeling. The results from these experiments are presented in Table 4.

**Table 4:** Experimental Results and Output Variables Calculated Based on Models^a^

no.	*x*_1_	*x*_2_	*x*_3_	*E/Z*	*E/Z*_cal_	DE	DE_cal_	DB	DB_cal_
1	0.8	130	3	0.46	0.46	0.79	0.79	11.1	11.1
2	1.2	130	3	0.70	0.74	0.73	0.72	15.3	15.2
3	0.8	170	3	2.80	2.75	0.87	0.88	15.9	16.0
4	1.2	170	3	gellation					
5	0.8	150	2	0.62	0.62	0.80	0.80	11.2	11.2
6	1.2	150	2	1.02	0.98	0.73	0.74	15.3	15.4
7	0.8	150	4	1.29	1.33	0.86	0.85	11.8	11.7
8	1.2	150	4	1.80	1.80	0.75	0.75	19.5	19.5
9	1.0	130	2	0.45	0.45	0.74	0.74	13.2	13.2
10	1.0	170	2	2.15	2.20	0.82	0.81	17.0	16.9
11	1.0	130	4	0.59	0.54	0.76	0.77	12.3	12.4
12	1.0	170	4	gellation					
13	1.0	150	3	1.24	1.21	0.79	0.78	15.4	14.2
14	1.0	150	3	1.3	1.21	0.79	0.78	14.0	14.2
15	1.0	150	3	1.09	1.21	0.77	0.78	13.3	14.2

a _cal_—calculated; *DE* [mgKOH/g]; *DB* [%].

Two reactions (no. 4 and 12) ended up gelling because of that analyses could not be done. Nevertheless, by applying the matrix equation, we obtained the mathematical equations which describe the synthesis process. Then, the statistical analysis of significance of the variables and the adequacy of the equations was done. All statistical data is presented in the Supporting Information All presented equations are calculated for coded variables.

**Figure 2:**
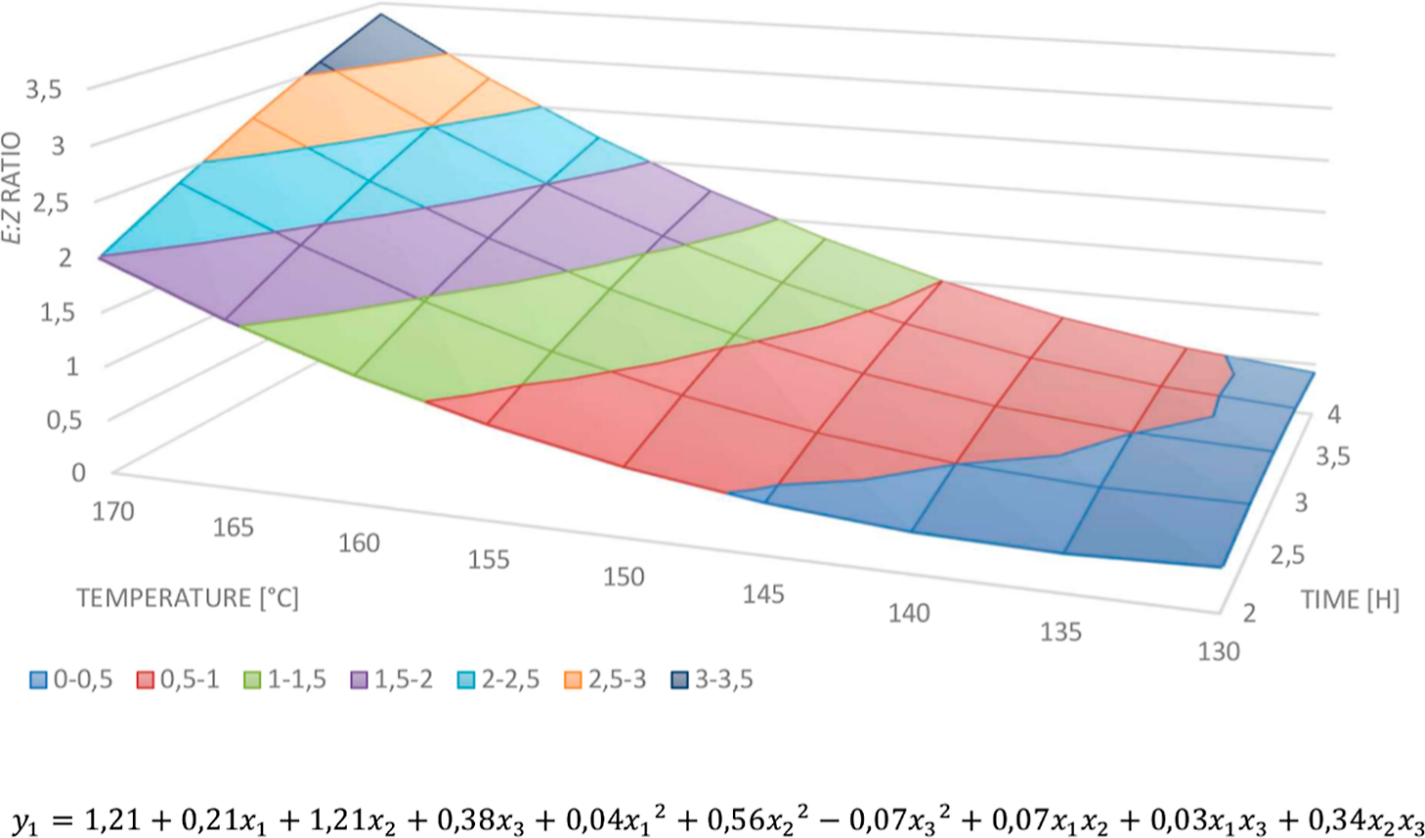
Response surface (maleic anhydride/glycerin ratio = 0.8) and model for determining the *E/Z* ratio.

The first model considered the *E*/*Z* isomer ratio. All the input variables, the temperature square, and the time and temperature interaction effect were significant to this output variable. The temperature and the time of the reaction were the most important. Moreover, their interaction effect had a stronger impact than the substrate molar ratio. Rejecting the insignificant monomials would not cause a better match of calculated and experimental results, so we decided not to reject any of them. The amount of E isomers increases with the growth of the temperature, the time, and the amount of the anhydride. The model and response surface are presented in Figure 2.

**Figure 3:**
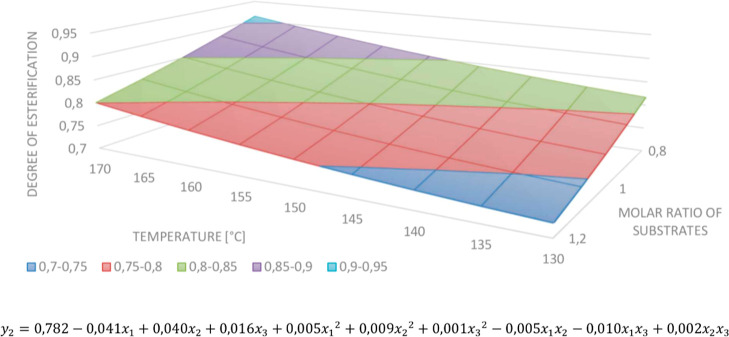
Response surface (the reaction time = 4) and model for determining *DE*.

The statistical analysis of the degree of the esterification model concludes that only the substrate molar ratio and the temperature are significant. According to the equation, the *DE* increases with the reduction of the amount of maleic anhydride and growth of the temperature. We observed neither the input variable interaction effect nor the significance of the variable squares. Nevertheless, rejecting the unimportant monomials did not cause better match of calculated and experimental results, so we decided not to reject any of them. The model and response surface are presented in Figure 3.

**Figure 4:**
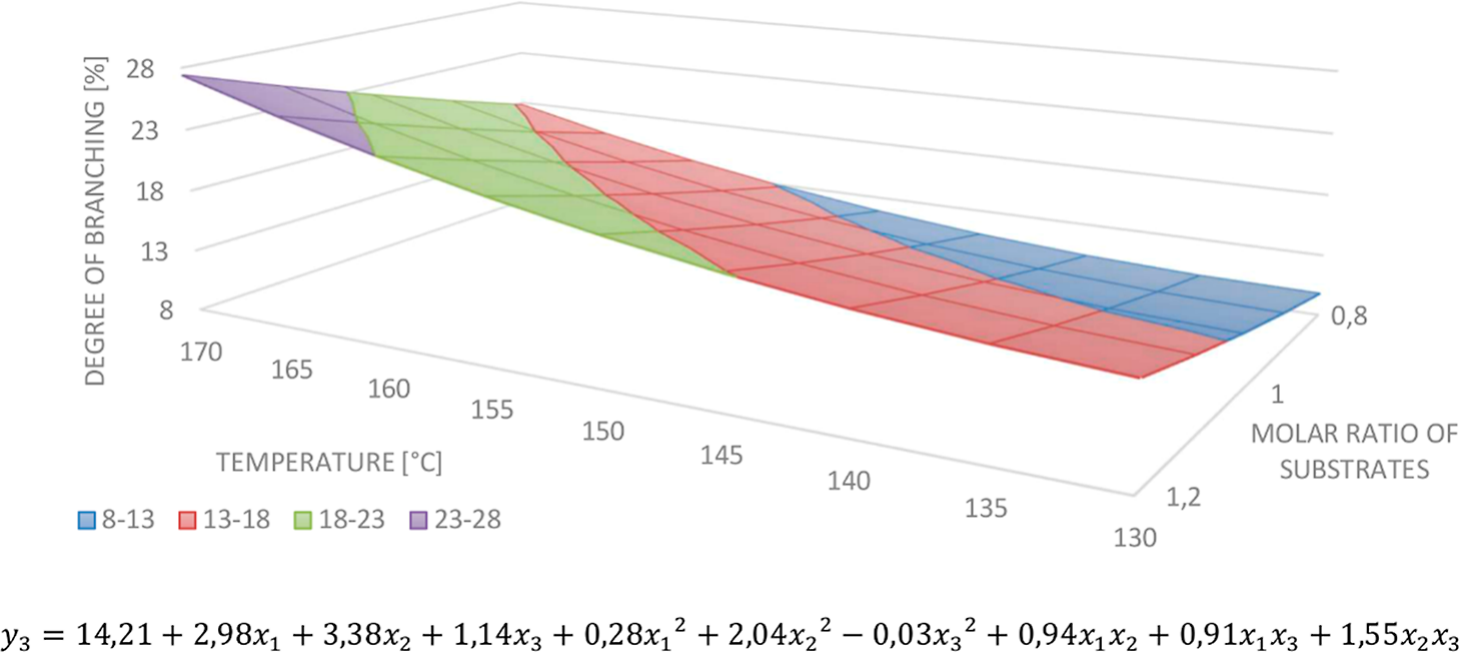
Response surface (the reaction time = 4) and model for determining *DB*.

In the case of the degree of branching, the substrate molar ratio and the temperature were the most relevant. According to the equation, the degree of branching increases with the growth of the temperature and the amount of the anhydride. We did not observe the relevance of the input variable interaction effect. Again, rejecting the insignificant monomials did not cause a better match of the calculated and experimental results, so we decided not to reject any of them. The model and response surface are presented in Figure 4.

### Optimal Reaction

According to the optimization criterium mentioned before, the optimal conditions were calculated using the Solver tool in MS Excel. The optimal conditions are *x*_1_—maleic anhydride/glycerin ratio = 0.8; *x*_2_—the temperature 130 °C, and *x*_3_—the reaction time 2 h. Then, the experiment was performed for checking and comparing the experimental and calculated results. It could be concluded that the mathematical equations obtained in the optimization describe the dependence of output variables on the input ones properly. The resulting polyester meets the goals set. Moreover, the developed synthesis was compared to an analogous one-step synthesis. That proves the relevance of the developed method of PGB synthesis. All results are presented in Table 5.

**Table 5:** Calculated and Experimental Results and Comparison to the One-Step Reaction^a^

variant	*E/Z*	*DE*	*DB*
optimal calculated	0.38	0.77	12.4
optimal experimental	0.49	0.79	9.22
one-step reaction	5.54	0.89	16.97

a *DE* [mgKOH/g]; *DB* [%].

## Conclusions

In this work, new polyester—poly(glycerin butenedioate) was obtained in the simple two-step bulk polycondensation of maleic anhydride and glycerin using neither catalysts nor solvents. Part of the Z double bonds transformed into E isomers, which were visible on ^1^H NMR spectra. The high temperature and longtime synthesis favored the isomerization.

Subsequently, the optimization of the synthesis was done with the use of the Box-Behnken plan. Using the matrix equations, the coefficients in the second-degree polynomials were calculated, showing how the output variables depend on the input ones. Three variables were tested on three levels: maleic anhydride-to-glycerin molar ratio (*x*_1_), the temperature (*x*_2_), and the time of reaction (*x*_3_). Determined mathematical models were statistically tested. These equations fit well to the experimental data and allow to calculation of optimal conditions. Optimization is also very important for industrial production in the future because it brings very valuable knowledge about the synthesis process.^21,70^

Determining the optimal conditions allow obtaining PBG dedicated for further unsaturated bond modification. For this reason, the obtained polyester should have a minimal *E/Z* isomer ratio and possible linear structure (*DB* less than 15%). The developed synthesis ensures such results. Of course, maximization of *DE* is important for obtaining polyesters with high molecular mass, but in this case, due to the possibility of cross-linking via an unsaturated bond reaction, is not crucial. As mentioned above, this work aims to develop the process for obtaining polyesters toward the best possibilities of unsaturated bond reactions.

Furthermore, considering glycerol polyesters’ still rising medical potential,^71−76^ PBG will probably be very attractive for medicinal uses. What is more, in our best knowledge, it is one of the first or even the first unsaturated glycerol polyester which could easily react on the α,β-unsaturated moiety. In this work, its structure was optimized for this purpose. Due to this reason, this material should find interest in scientists researching DDSs and injectable (noninvasive) scaffolds for tissue regeneration.

## Supporting Information Available

Description of preliminary studies, HSQC spectra, and statistical data (PDF)

## Abbreviations

PGB: poly(glycerol butenedioate)
*E*: *Z*/E isomer-to-Z isomer ratio
*DE*: degree of esterification calculated from acid and ester number
*DB*: degree of branching
