# Supplementary material for: Synthesis of Poly(glycerol butenedioate)—PGB—Unsaturated Polyester toward Biomedical Applications
Source: ACS Omega. 2022 Jul 15;7(29):25171–8. doi: 10.1021/acsomega.2c01934 (PMC9330079; doi:10.1021/acsomega.2c01934)
Supplement: Supplementary file 1 — ao2c01934_si_001.pdf [file ao2c01934_si_001.pdf]

Synthesis of poly(glycerol butenedioate) – PGB – unsaturated polyester towards biomedical applications

Supporting Information

*Michał Wrzecionek[1], Krzysztof Kolankowski[1], Agnieszka Gadomska-Gajadhur[1]**

[1]Faculty of Chemistry, Warsaw University of Technology, 3 Noakowskiego Street, Warsaw, 00-664, Poland

Preliminary studies description

In the beginning, the reactions were carried out in the one-stage variant at different conditions (**Table S1**). The input variables were x1 – maleic anhydride to glycerin molar ratio, x2 – the temperature and x3 – the time of reaction and were the same as in our previous, published optimization.21

We carried out the reactions:

A – in the argon flow,

B – at high temperature, collecting water in the Dean-Stark apparatus,

C – under reduced pressure,str. 2

D – with cyclohexane as an azeotropic agent,

E – without water collection.

Table S1 Preliminary studies of polycondensation; x_1-maleic anhydride to glycerin molar ratio, x_2-the temperature, x_3-the time of reaction, $\%_{H2O}$-water content, *DE*-the degree of esterification

Nr	x_1	x_2 [˚C]	x_3 [h]	*DE*	variant
1	2:3	170	4	0,84	E
2	2:3	170	4	0,93	A
3	2:3	170	24	-	B
4	2:3	85	24	0,79	D
5	2:3	150	4	0,84	C

6	2:3	150	24	0,93	E+C
7	1:1	150	4	0,75	E
8	2:3	150	4	0,84	E
9	2:1	150	4	-	E

It is worth noting that applying the argon flow (2) was the most successful method to collect water. Unfortunately, we observed the resublimation of white residue outside the reactor and the acidic pH of the collected liquid, which means that part of anhydride and acidic esters were removed from the reactor. A slightly less successful method was reducing the pressure in the reactor (5). By comparing it to the reaction carried out at the same conditions but without reducing pressure, we obtained a product with the same degree of esterification (8). Moreover, by reducing the pressure, we also observed removing anhydride and acidic esters. By mixing these two variants, which means reducing pressure only at the end of the process we obtained product with high degree of esterification and eliminated the problem of resublimation of reagents outside the reactor (6). We also found out that even though the temperature was higher than 100˚C, the water did not

appear in the Dean-Stark apparatus (7, 8, 9). Water was collected only if the temperature in the reactor was ca. 170˚C (3). But maintaining such a high temperature for many hours and collecting water ended up with gelling of the product so its characterization was impossible. Using cyclohexane as an azeotrope agent was the unsuccessful method of water collection (4).

We also carried out two experiments at which different substrates molar ratios were used: equimolar ratio (7) and anhydride excess (9). In the second case (9) we observed gelling of the product after 4 hours of the reaction.

Consequently, we decided to carry out the reaction without water collection at the beginning and later to reduce pressure to remove water from the reactor.

NMR spectrums

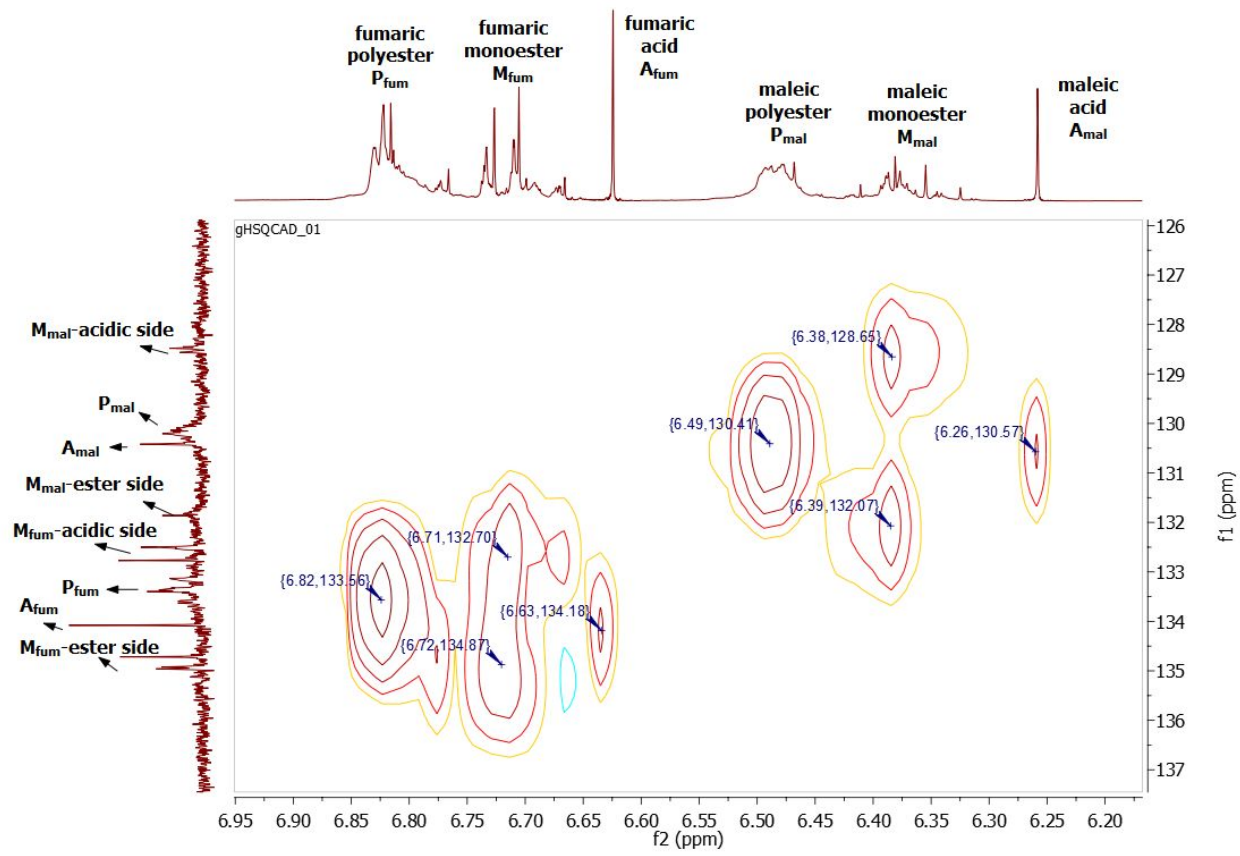

Figure S1 HSQC spectrum with signals interpretation at range characteristic for acid part

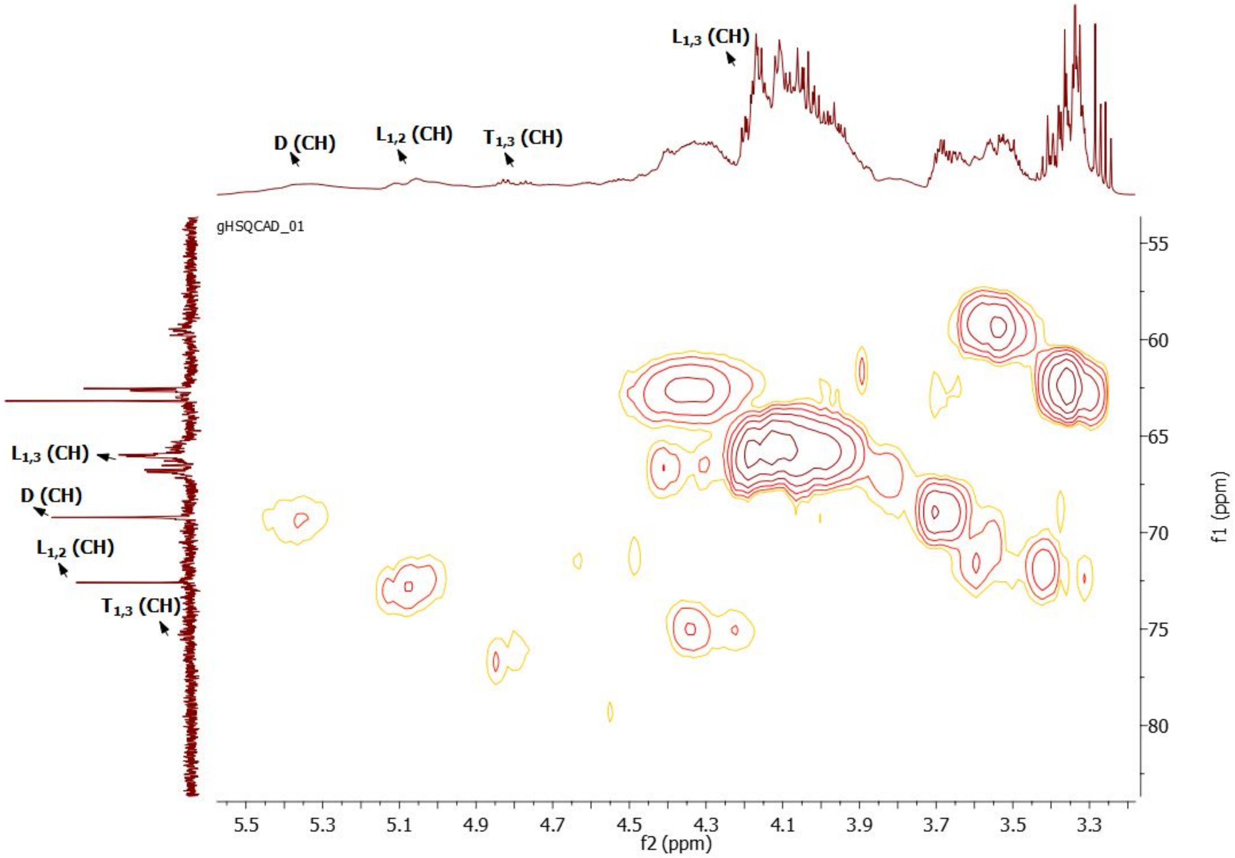

Figure S2 HSQC spectrum with signals interpretation at range characteristic for glycerol part

Statistical data

Table S2 Significance of coefficients of the equation for the *E:Z* isomers ratio

factor	coefficient estimate (bj)	standard error (sbj)	critical value t =4,31	
			t value	significance

b0	1,21	0,062	19,520	yes
b1	0,21	0,038	5,405	yes
b2	1,21	0,038	31,890	yes
b3	0,38	0,038	10,112	yes
b11	0,04	0,107	0,363	no
b22	0,56	0,107	5,239	yes
b33	-0,07	0,107	0,610	no
b12	0,07	0,054	1,212	no
b13	0,03	0,054	0,510	no
b23	0,34	0,054	6,303	yes

Table S3 Significance of coefficients of the equation for the degree of esterification

factor	coefficient estimate (bj)	standard error (sbj)	critical value t =4,31	
			t value	significance
b0	0,782	0,0086	90,703	yes
b1	-0,041	0,0053	7,681	yes
b2	0,040	0,0053	7,578	yes
b3	0,016	0,0053	3,096	no
b11	0,005	0,0149	0,324	no
b22	0,009	0,0149	0,622	no
b33	0,001	0,0149	0,065	no
b12	-0,005	0,0075	0,691	no
b13	-0,010	0,0075	1,305	no
b23	0,002	0,0075	0,307	no

Table S4 Significance of coefficients of the regression equation for the degree of branching

factor	coefficient estimate (bj)	standard error (sbj)	critical value t =4,31	
			t value	significance
b0	14,21	0,605	23,502	yes
b1	2,98	0,370	8,037	yes
b2	3,38	0,370	9,126	yes
b3	1,14	0,370	3,088	no
b11	0,28	1,047	0,267	no
b22	2,04	1,047	1,947	no
b33	-0,03	1,047	0,026	no
b12	0,94	0,524	1,794	no
b13	0,91	0,524	1,733	no

b23	1,55	0,524	2,958	no
